# Ruminal Degradability and Summative Models Evaluation for Total Digestible Nutrients Prediction of Some Forages and Byproducts in Goats

**DOI:** 10.1155/2013/532528

**Published:** 2013-05-13

**Authors:** Oswaldo Rosendo, Luis Freitez, Rafael López

**Affiliations:** ^1^Departament of Animal Nutrition and Forages, Decanato de Ciencias Veterinarias, Universidad Centroccidental Lisandro Alvarado, Tarabana 3001, Venezuela; ^2^Universidad del Zulia, Maracaibo 4005, Venezuela

## Abstract

In in vitro true dry matter degradability (IVTDMD), in situ dry matter degradability, and neutral detergent fiber degradability, both in vitro (IVNDFD) and in situ (ISNDFD) techniques were used with crossbred goats to determine dry matter and neutral detergent fiber (NDF) ruminal degradability in eight forages and four industrial byproducts. Total digestible nutrients (TDN) content obtained with five different summative models (summative equations) were studied to compare the precision of estimates. All these models included digestible fractions of crude protein, ether extract, and nonfiber carbohydrates that were calculated from chemical composition, but digestible NDF (dNDF) was obtained from IVNDFD (IVdNDF), ISNDFD (ISdNDF), or by using the Surface Law approach. On the basis of the coefficient of determination (*R* ^2^) of the simple lineal regression of predicted TDN (*y*-axes) and observed IVTDMD (*x*-axes), the precision of models was tested. The predicted TDN by the National Research Council model exclusively based on chemical composition only explains up to 41% of observed IVTDMD values, whereas the model based on IVdNDF had a high precision (96%) to predict TDN from forage and byproducts fiber when used in goats.

## 1. Introduction

Currently, feeding standards for ruminants are based on predictions of total digestible nutrients (TDN), digestible energy (DE), metabolizable energy (ME), and/or net energy (NE) from the in vitro digestibility determinations of organic matter (IVOMD) and/or chemical composition [1–3]. However, there are no energy prediction equations created and validated specifically for goats. The tabulated energy values in the National Research Council (NRC) for small ruminants [4] are based on measurements of TDN in vivo, most of which were carried out some years ago, in sheep. Therefore, the prediction of TDN is justified to increase the accuracy of the values used in ration formulation and ultimately reduce the risks of energy imbalances in dairy goats.

There are a number of prediction equations in ruminants, from simple regression equations [5–7] to equations called “multiple” or “summative” [8, 9]. However, it is recommended to evaluate the validity of any prediction equation by taking into account the evaluation of digestibility and chemical composition of local feedstuff [2].

The summative equation developed by Weiss et al. [9] and later adopted by the NRC (2001) of dairy cattle [3] calculated each digestible feed fraction and among these, the potentially digestible NDF (dNDF) by a subecuación which is based on the nonlinear relationship between NDF and lignin. This relationship implies that lignin has a physical effect, besides the chemical effect of protection against digestion of structural carbohydrates, making the NDF indigestible [10, 11]. Theoretically, the indigestible NDF is proportional according to the surface law to two-thirds of the surface area represented by the mass of lignin, but inversely proportional to the surface of NDF [10, 12]. However, the prediction of the energy value can be a problem for tropical forages and fibrous feed, due to the high variability in NDF digestibility (NDFD), as well as to the low predictive power that the chemical composition has on NDFD [13]. This limitation could be overcome in goats if the potentially digestible neutral detergent fiber fraction is determined by methods in situ or in vitro.

Adapting dairy goat diets to high levels of fiber is well documented [14–16]. However, the evaluation of ruminal degradability, in situ or in vitro, in forages and byproducts is rare when it comes to goats. Although forage DM and NDF digestibility can be similar for cattle and goats under maintenance [17]; the equation of NRC (2001) [3] should not be extrapolated to dairy goats, without prior evaluation, due to known differences that occur between these two species in their level of intake, passage rate, and retention time [16–18].

The present study was aimed to determine the ruminal degradability of DM and NDF, both in vitro and in situ, on forages and fibrous byproducts using ruminal liquor of goats and second to evaluate the usefulness of ruminal degradability of fiber together with chemical composition to predict the TDN content. More specifically, different summative models that use digestible NDF fraction to predict TDN are evaluated. 

## 2. Materials and Methods

Eight samples of forages and 4 byproducts were received at the laboratory of Animal Nutrition, College of Veterinary Science (University Lisandro Alvarado, Venezuela), for routine chemical analysis. Samples were selected based on obtaining sufficient variabilidad both in chemical composition and ruminal degradability. These samples were bermudagrass (*Cynodon dactylon*), stargrass (*Cynodon nlemfuensis*), guinea grass (Panicum maximum), king grass (*Pennisetum purpureum*, cv king grass), Kikuyu grass (*Pennisetum clandestinum*), Brachiaria (Brachiaria decumbens), forage sorghum (Sorghum bicolor), sugar cane and byproducts (discarded corn, *Zea mays*; discarded sorghum, *sorghum vulgare*; discarded sesame, Sesamum indicum and citrus pulp, *citrus sp*.). The crude protein content ranged from 5.2 to 10.3% in the byproducts and 4.3 to 17.7% in forages. While NDF content ranged from 25.9 to 77.9% with the highest values in forages and the lowest in byproducts. Meanwhile, the value of NDICP ranged from 1.2 to 5.8% in byproducts, while in forages ranged from 3.2 to 8.8% (Table 1).

### 2.1. Chemical Analyzes

The samples were dried at 100°C in an oven for 24 h to determine DM and then ground to 1 mm in a Wiley mill. Crude protein analysis, ADF, and Ash were performed according to the procedures of the Association of Official Chemical Analysis (AOAC) [19]. The NDF was determined using amylase and sodium sulfate [20]. Insoluble crude protein concentrations in ADF (ADICP) and NDF (NDICP) were analyzed according to procedures described by Licitra et al. [21]. Lignin (L) was quantified according to the sulfuric acid method [19]. The nonfiber carbohydrate concentration (CNF) was calculated according to the following equation created by Nocek [20] and described by Van Soest et al. [22]:

(1) NFC,% =100−[CP%+(NDF%−NDICP%)+EE%+Ash%],

where NICP = Neutral detergent insoluble crude protein, EE = ether extract.

### 2.2. Animals and Diet

Three nonlactating and non-pregnant cross-bred adult goats (mean body weight = 43 kg) were fistulated in the rumen for using as donors of ruminal inoculum. Rubber cannulae (80 g weight, 35 mm internal diameter) were fabricated locally. The surgical technique consisted of a straight incision of equal length to the diameter of the cannula, without fasting, standing up during the surgical procedure [23]. The goats were used 12 weeks after surgery. Animals received a ration consisting of ad libitum bermudagrass hay (7.7% CP, 71.8% NDF, and 51.8% TDN, on dry basis) and 500 g/day of commercial supplement (18.4% CP, 33.2% NDF, and 67.2% TDN, dry basis). The ration was provided during an adjustment period of 20 days.

### 2.3. In Vitro Incubations

One in vitro incubation was performed within each period (*n* = 3) of 21 days. On day 16 of each period, ruminal fluid was collected from the three goats after 15 days of adaptation to the diet, filtered through four layers of cheesecloth, mixed, and then carried out to the laboratory in a thermo. In vitro true DM digestibility (IVTDMD) and in vitro NDF digestibility (IVNDFD) was determined by the technique described by Van Soest et al. [24]. The in vitro system consisted of 50 mL polypropylene tubes (Fisher Scientific., USA) fitted with rubber stoppers and Bunsen valves (Fisher Scientific., USA). Incubations (Thelco Incubator, Precision Scientific., USA) were performed serially for 48 and 72 hours at 39°C, for a total of 104 tubes ((twelve feed + blank) × 2 tubes/sample × 2 digestibilities × 2 times), in each period. The tubes were gently shaken after incubation and at 6 hours intervals. Blank tubes (2) contained inoculum but no substrate was placed in each incubation time to estimate the amount of DM and NDF supplied by the inoculum in order to make corrections.

### 2.4. In Situ Incubations

 Triplicate samples from each feed were placed and weighed (3 g) in nylon bags (Ankom) adjusted to a size of 10 × 10 cm. A total of 36 bags (twelve feed) were placed inside six pantyhose (6 feed/pantyhose). After immersed in distilled water at 39°C for 5 min., a pantyhose was incubated in the rumen of each animal for 48 h, at day 17 and 19 of each period. After, removal from the rumen, the bags were manually washed and dried for 48 hours at 60°C.

### 2.5. Prediction of Total Digestible Nutrients

To estimate TDN at maintenance levels (NDT_1*X*_), the following equations from NRC (2001) [3] were used:

(2) TDN1X=dCP+(dFA∗2.25)+dNFC+dNDF−7,dCP(forages)=CP∗EXP(−1.2∗(ADICPCP)),dCP(concentrates)=CP∗(1−(0.4∗(ADICPCP))),  dFA=1.00∗(EE−1),dNFC=FAP∗0.98∗(100−(Ash+CP+EE  +(NDF−NDICP))),

where TDN_1*X*_ = total digestible nutrients at intake levels of maintenance, dCP = digestible CP, dFA = digestible fatty acids, dNFC = digestible nonfiber carbohydrates, FAP = Adjustment factor due to processing, ADICP = acid detergent insoluble crude protein, NDICP = neutral detergent insoluble crude protein (all expressed on a dry basis), and 7 represents endogenous losses of TDN. For all feed, FAP was 1.0 (Table 2.1 NRC 2001) in [3], while to calculated CP, byproducts were considered as concentrates.

The dNDF was calculated either from the IVNDFD (IVdNDF), ISNDFD (ISdNDF), or as a function of the surface law (LSdNDF) according to models that are explained below.

(a) LSdNDF (dNDF calculated based on the law of surface): based on the original equations of NRC (2001) [3] for calculating TDN exclusively from the chemical composition and wherein the dNDF is related to lignin concentration using a function based on the law of surface:
  (3) dNDF=0.75∗((NDF−NDICP)−L)∗(1−(L(NDF−NDICP))0.667).
(b) IVdNDF (100% of dNDF calculated from IVNDFD): this was based on using the technique of in vitro degradability during 48 hours of fermentation to calculate dNDF as an alternative manner proposed by NRC (2001) [3]:
  (4) IVdNDF=1.00∗(IVNDFD100)∗NDF,%.
(c) 0.75IVdNDF (75% of dNDF calculated from IVNDFD): it was based on the computation of the dNDF using the technique of in vitro degradability for 48 hours of fermentation but multiplying by 0.75 (0.75IVdNDF), equivalent to the digestibility of potentially digestible NDF fraction according to the original equation of NRC (2001) [3]:
  (5) 0.75IVdNDF=0.75∗((IVNDFD100)∗NDF,%).
(d) ISdNDF (100% of dNDF calculated from ISNDFD), calculation of dNDF was based on in situ degradability of NDF during 48 hours of fermentation, thus
  (6) ISdNDF  =  1.00∗(ISNDFD100)∗NDF,%.
(e) Rocha-Junior (dNDF calculated as Rocha-Junior): TDN was calculated using the NRC model [3] modified in Brazil by Rocha-Junior et al. [25] where dNDF equation was adjusted by nonlinear regression between observed and calculated values from subecuación of NRC (2001) [3]. The adjusted coefficient for dNDF is given by the following equation [25]:
  (7) Adjusted  dNDF=0.6232∗((NDF−NDICP)−L)∗(1−(L(NDF−NDICP))1.2258).

The rest of the equations to calculate TDN_1*X*_ were similar to NRC (2001) [3].

### 2.6. Statistical Analysis

The study was analyzed as two different experiments (one in vitro and one in situ) with three replications for each. In the in vitro experiment, degradability (48 or 72 h) of DM or NDF (*Yijklm*) of each feed (*A*) within each repetition (*R* = 3) was analyzed according to the following model:

(8) Yijk=m+Ai+Rj+ARIJ+eijk.

In the in situ experiment, degradability of DM or NDF at 48 h (*Yijklm*) for each feed (*A*) within each repeat (*R* = 3) was arranged by goat (*C* = 3), according to following model:

(9) $$\begin{array}{c} Yijklm=m+Ai+Rj+Ck+ARIJ+ARCijk+eijkm. \end{array}$$

All the analyses were performed by using PROC GLM of SAS [26]. Averages for IVTDMD, IVNDFD, and ISNDFD were compared using the method of minimum significant differences (*P* < 0.05). 

### 2.7. Quality Assessment of Summative Equations to Predict TDN

The evaluation of the accuracy of the model (summative equation) to estimate TDN was performed using the coefficient of determination (*R* ^2^) of the linear regression of the predicted values of NDT for the model and observed values of IVTDMD, using PROC REG in SAS [26]. We chose this relationship (TDN and IVTDMD) to evaluate different summative equations for TDN, because in forages, the IVTDMD has been an acceptable predictor (*R* ^2^ = 0.90) of the in vivo digestibility of DM in sheep [27]. Moreover, there are no distinct differences between sheep and goats, as for passage rate, retention time, and digestibility, when they are fed at maintenance conditions [28, 29].

## 3. Results and Discussion 

### 3.1. In Vitro True Dry Matter Degradability and In Situ Dry Matter Degradability

In vitro true dry matter degradability and in situ dry matter degradabilityvalues of IVTDMD at 48 and 72 h of incubation are shown in Table 2, whereas ISDMD at 48 h are shown in Table 3. With the exception of extensive degradation of MS in citrus pulp [30], the values of DM degradability were higher, intermediate, and lower for 72 h IVTDMD, and 48 h ISDMD, 48 h IVTDMD, respectively. DVIVMS values were higher at 72 h, up to 15%, to those found at 48 h.

In forages, the IVTDMD at 48 h ranged from 51.4 to 80.8%, with the lowest value for Brachiaria and the highest for the stargrass at 20 days of age (*P* < 0.05). High IVOMD values are often seenfor preflowering stargrass with high levels of nitrogen fertilization [31]. However, this study was not specifically designed to compare the ruminal degradability among different forage species. In byproducts, IVTDMD at 48 h had the range of 68.1 to 77.4%, with the highest value for discarded corn.

The chemical composition and IVTDMD of bermudagrass were consistent with those reported by other researchers [32]. The results suggest that those forages with higher CP and lower NDF concentration, as was the case of Guinea grass and stargrass, can be digested with greater efficiency by goats compared to bermudagrass. Therefore, the replacement of commercial bermuda hay, main source of forage for dairy goats in Venezuela, by other forage substitutes with greater DM degradability would maximize milk production in confined systems.

The ISDMD at 48 h was highly related to IVTDMD at 48 h (*R* ^2^ = 0.92) but with values up to 20% lower. Therefore, these results suggest that values of ISDMD at 48 h may underestimate in vivo DM digestibility.

### 3.2. In Vitro and In Situ Neutral Detergent Fiber Degradability

The values of IVNDFD at 48 and 72 h of incubation are shown in Table 2. An in vitro incubation period of 48 h may be appropriate to approach the in vivo digestibility values of NDF for dairy goats, because ruminal retention time for 10 hays of perennial grasses, including Bermuda, varied in the range of 24.9 to 53.3 h in goats under maintenance conditions [13].

In the present study, IVNDFD at 48 h ranged from 36.9 to 72.8% for forages with the highest value for stargrass and the lowest for Brachiaria, which explains the observed IVTDMD results. Thus, the lower NDF digestibility values observed were those forages with lower CP and higher ADICP contents; suggesting that microbial digestion of NDF was limited by the low availability of fermentable N. In goats, in vivo NDF digestibility of grass tends to reach values close to 70% when the CP content is 15% but only 60% or less when the concentration of CP lower than 10% [33–35].

In the byproducts evaluated, the IVNDFD had the range from 4.8 to 80.5%, with the lowest values for discarded sesame and discarded sorghum. The possible causes of high NDF indigestibility for discarded sesame may be associated with high fat and cutin contents [4, 11].

**Figure 1:**
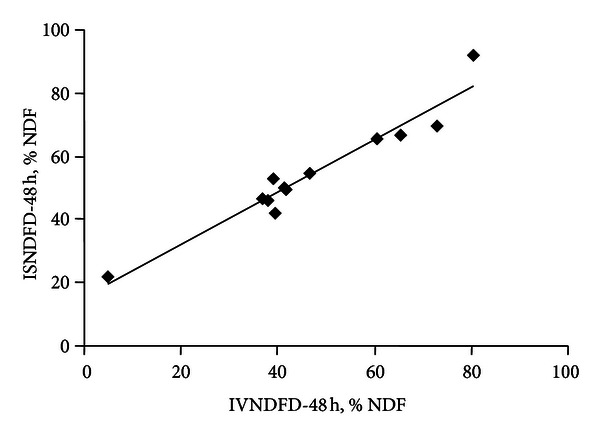
Relationship between the in vitro (*X*) and the in situ (*Y*) degradability of NDF. *Y* = 0.8313*X* + 15.475. *R* ^2^ = 0.94.

In general, the IVNDFD values at 48 h (Table 3) were lower than the values of ISNDFD at 48 h, particularly for sesame discarded and citrus pulp; however, they were highly correlated. Thus, IVNDFD explained 94% of the variability in ISNDFD values (Figure 1). In cattle, Spanghero et al. [36] found a similar relationship, but on the contrary, the IVNDFD values were on average 19% higher than the effective in situ degradability of NDF. Robinson et al. also reported higher IVNDFD than ISNDFD values at 48 h [37].

### 3.3. Prediction of Total Digestible Nutrients

Some researchers have used IVTDMD or IVOMD values to calculate the values of TDN in forages and supplements when it comes to cattle [38, 39]. The method of Tilley and Terry [40] as well as that of IVTDMD of Van Soest et al. [24] that replaces the 48 hours of pepsin incubation with extraction on neutral-detergent solution has a high correlation with the in vivo DM digestibility under maintenance conditions [27, 41].

The predicted TDN values obtained by evaluated summative equations are shown in Table 4. Regression parameters between the predicted and observed values were calculated by using IVTDMD 48 h as the accepted observed values (Table 5). In this study, TDN values predicted, both by the original equation of NRC dairy cattle [3], based exclusively on chemical composition (LSdNDF), as well as by the modified model of Rocha-Junior et al. [25], were less successful than those predicted by the equations that incorporate dNDF determined by either in vitro (IVNDFd) or in situ (ISNDFd) digestibility. Conversely, Magalhães et al. [42] found that models based on dNDF determined in vitro or in situ underestimated ME values for cattle when compared to the original NRC model based on chemical composition.

The NRC model to calculate TDN (LSdNDF) is based on the calculation of dNDF as a function of the surface law. While lignin raised to 0.66 (according to the law of surface) can explain most linear effects that L and FND have in dNDF, this does not include all possible determinants of dNDF [43]. The 0.85 exponent instead of 0.66 can improve the prediction accuracy of the NDF indigestible fraction from lignin content [2, 13]. Therefore in tropical feed, the restrictive effect of lignin in NDF degradation is less intense than that proposed by the law of surface [2].

Advantageously, the in vitro fermentation can include the impact of factors other than lignin on ruminal fiber degradation, for example, effects of nonstructural carbohydrate, fat, silica, or cutin [11, 44]. Furthermore, it is difficult to get accurate values of lignin by gravimetric analyses, since lignin values varies depending on the analytical method used [45].

The NRC (2001) [3] suggests using IVdNDF values to replace the use of lignin in the calculation of TDN. Similarly, Shaver [8] used IVdNDF in a summative equation to calculate the EN_*L*_ value corn (Zea mays) silage. Using bovine rumen fluid, Lundberg et al. [39] compared the values of predicted TDN based on IVdNDF versus in vitro digestible organic matter (IVdOM) values as acceptable predictors of in vivo OM digestibility in cattle. In that study, IVdNDF explained 98% of the observed variability in IVdOM values of corn silage.

The in situ degradability technique for determining dNDF has also been used in the calculation of TDN and other energy systems values for dairy cows [33].

The results of the present study also suggest that the 0.75 coefficient used for dNDF in the NRC [3] equation is inappropriate as noted by several researchers [12, 17]. Similarly, the adjusted coefficients for dNDF according Rocha-Junior et al. [25] were inappropriate.

### 3.4. Energy Calculations at Production Levels

By definition, TDN corresponds to ingestion levels of maintenance (NDT_1*X*_) but Sutton and Alderman [18] indicate that high-producing lactating goats can reach intakes equivalent to 3 to 4 times maintenance needs. Therefore, both the digestibility and TDN value of the diet is reduced. In dairy cows, the digestibility of good quality diets is reduced by approximately 8% when the DM intake levels reach up to three times the maintenance needs [3]. Therefore, the values of TDN in lactating goats, with intakes of up to 3 times the maintenance level (NDT_3X_), can be estimated in mixed diets following the NRC procedure for dairy cows [3] as follows.

 If TDN_1*X*_ < 60% : TDN_3*X*_ = TDN_1*X*_
 If TDN_1*X*_ > 60% : TDN_3*X*_ = TDN_1*X*_ − (0.18∗TDN_1*X*_ − 10.3)∗2.

In turn, the concentration of ME at production levels (3 times the maintenance level) could be calculated using the following standard equations [46]:

(10) $$\begin{array}{c} {\text{ME}}_{3X},\text{Mcal}/\text{kg}=0.036\times {\text{TDN}}_{3X}. \end{array}$$

Table 6 shows the conversion of TDN_1*X*_ values, calculated using IVdNDF into ME values at production levels.

## 4. Conclusions

In general, values of IVTDMD at 48 h were higher than ISDMD, except for citrus pulp. In forages, IVTDMD had a rank (51.4 to 80.8%) greater than in byproducts (68.1 to 94.9%). Values for ISNDFD and IVNDFD at 48 h were highly correlated (*r* = 0.94). The NDF of discarded sesame was highly indigestible. The TDN values predicted by the NRC model, exclusively based on chemical composition, explained only 41% of the observed variability in IVTDMD values; therefore, they were less successful than those predicted by models incorporating dNDF determined by either in vitro or in situ digestibility. In vitro NDF digestibility is an acceptable method to estimate the dNDF fraction in summative equations. However, the more accurate model was that where the value of dNDF is used with a coefficient equal to 1.

## Figures and Tables

**Table 1:** Chemical composition of evaluated feed.

Feed	DM %								
	OM	CP	EE	NDF	ADF	L	NDICP	ADICP	NFC^1^
Discarded corn	92.4	10.3	2.4	57.3	21.0	6.7	5.8	3.3	28.2
Discarded sorghum	93.1	7.9	2.2	43.9	23.1	6.8	5.5	2.1	44.5
Discarded sesame	73.7	5.2	13.6	32.8	50.8	6.8	4.9	2.6	26.9
Citrus pulp	94.9	6.1	3.1	25.9	28.9	6.4	1.2	1.3	61.0
Sugar cane, whole plant, 6 months	96.3	4.3	0.8	63.9	38.6	6.5	3.2	2.0	30.5
Forage sorghum	93.1	5.3	0.9	68.9	46.4	9.0	5.5	3.3	23.5
Bermudagrass	90.3	8.0	1.0	77.9	46.5	6.6	6.0	2.6	9.3
Brachiaria	95.8	7.1	1.6	77.0	41.9	8.7	5.7	2.9	15.8
King grass	93.1	5.8	1.6	73.0	47.0	5.9	3.9	2.2	16.7
Kikuyu grass	85.2	7.4	0.8	73.2	42.8	7.8	5.4	2.0	9.1
Guinea grass	87.1	12.7	1.9	71.6	46.3	8.3	7.1	1.7	8.0
Stargrass, 20 days	90.8	17.7	1.4	70.5	40.0	6.1	8.8	1.8	9.9

^1^NFC, %: 100 − [CP% + (NDF%  −  NDICP%) + EE% + Ash%], where, NDICP: neutral detergent insoluble crude protein, EE: ether extract.

**Table 2:** Means and standard errors of the in vitro true DM digestibility (IVTDMD) and in vitro NDF degradability (IVNDFD) at 48 and 72 h of incubation.

Feed	IVTDMD-48	IVTDMD-72	IVNDFD-48	IVNDFD-72
Discarded corn	77.4^cd^	81.6^d^	60.6^cd^	67.9^c^
Discarded sorghum	73.2^d^	79.1^d^	39.0^fg^	52.5^d^
Discarded sesame	68.1^e^	70.3^e^	4.8^h^	9.5^e^
Citrus pulp	94.9^a^	96.6^a^	80.5^a^	86.9^a^
Sugar cane, whole plant, 6 months	62.8^f^	67.4^ef^	41.8^fg^	49.0^d^
Forage sorghum	58.2^fg^	65.5^f^	39.3^fg^	50.0^d^
Bermudagrass	54.3^g^	62.4^fg^	41.4^fg^	51.7^d^
Brachiaria	51.4^g^	59.1^g^	36.9^g^	46.8^d^
King grass	54.7^g^	62.2^fg^	38.0^fg^	48.3^d^
Kikuyu grass	61.0^f^	65.1^f^	46.7^ef^	52.4^d^
Guinea grass	75.2^d^	80.8^d^	65.6^bc^	73.4^bc^
Stargrass, 20 days	80.8^c^	85.9^c^	72.8^ab^	80.1^ab^
Standard error	1.6	1.3	3.1	3.4

a,b,c,d,e,f,g, means within a column with different superscripts differ (*P* < 0.05).

**Table 3:** Means and standard errors for in situ DM (ISDMD) and NDF degradability (ISNDFD) in nylon bag at 48 h of incubation.

Feed	ISDMD-48	ISNDFD-48
Discarded corn	65.5^d^	65.3^b^
Discarded sorghum	67.3^d^	52.6^c^
Discarded sesame	57.0^e^	22.1^f^
Citrus pulp	96.4^a^	92.0^a^
Sugar cane, whole plant, 6 months	47.1^fg^	42.1^de^
Forage sorghum	47.5^fg^	50.1^c^
Bermudagrass	45.9^fg^	46.6^cd^
Brachiaria	58.4^e^	49.3^cd^
King grass	46.0^fg^	46.1^cd^
Kikuyu grass	49.1^f^	54.6^c^
Guinea grass	64.5^d^	66.8^b^
Stargrass, 20 days	67.7^d^	69.8^b^
Standard error	1.6	2.7

a,b,c,d,e,f,g, means within a column with different superscripts differ (*P* < 0.05).

**Table 4:** Predicted TDN values (%) from summative models.

Feed	LSdNDF	0,75IVdNDF	IVdNDF	ISdNDF	Rocha-Junior
Discarded corn	57.7	58.8	67.5	70.2	58.3
Discarded sorghum	62.7	59.3	63.6	69.6	638
Discarded sesame	61.7	53.1	53.5	59.2	62.8
Citrus pulp	71.3	78.8	84.0	87.0	72.4
Sugar cane, whole plant, 6 months	56.8	45.4	52.1	56.9	56.9
Forage sorghum	48.3	38.9	45.6	47.6	49.4
Bermudagrass	46.7	31.7	39.8	46.6	46.1
Brachiaria	49.6	35.5	42.6	50.0	50.2
King grass	52.5	35.1	42.1	48.0	51.7
Kikuyu grass	41.6	32.9	41.5	47.3	42.0
Guinea grass	45.2	48.9	60.7	61.5	45.9
Stargrass, 20 days	52.2	57.9	70.7	68.6	52.0

**Table 5:** Effect of the TDN prediction model on the relationship between TDN predicted value and the observed value of IVTDMD at 48 h.

Model	Intercept	Slope	*R* ^2^
LSdNDF	+25.543	0.4183	0.41
0,75IVdNDF	−22.009	1.0362	0.90
IVdNDF	−16.169	1.0576	0.96
ISdNDF	−3.246	0.9254	0.91
Rocha-Junior	+24.606	0.4391	0.42

**Table 6:** Calculated TDN values at maintenance conditions using equation IVdNDF from NRC [3] and its conversion to ME values at production levels.

Feed	TDN_1*X*_	TDN_3*X*_	ME_3*X*_
	%	%	Mcal/kg DM
Discarded corn	67.5	63.8	2.4
Discarded sorghum	63.6	61.3	2.2
Discarded sesame	53.5	535	1.9
Citrus pulp	84.0	74.4	2.8
Sugar cane, whole plant, 6 months	52.1	52.1	1.8
Forage sorghum	45.6	45.6	1.5
Bermudagrass	39.8	39.8	1.3
Brachiaria	42.6	42.6	1.4
King grass	42.1	42.1	1.4
Kikuyu grass	41.5	41.5	1.4
Guinea grass	60.7	59.4	2.2
Stargrass, 20 days	70.7	65.9	2.5

If TDN_1*X*_ < 60%: TDN_3*X*_ = TDN_1*X*_. If TDN_1*X*_ > 60%: TDN_3*X*_ = TDN_1*X*_  − (0.18 ∗ TDN_1*X*_  − 10.3) ∗ 2. ME_3*X*_, Mcal/kg = 0.036  ×  TDN_3*X*_.
